# Interleukin-13 increases the stemness of limbal epithelial stem cells cultures

**DOI:** 10.1371/journal.pone.0272081

**Published:** 2022-08-02

**Authors:** Peter Trosan, Joao Victor Cabral, Ingrida Smeringaiova, Pavel Studeny, Katerina Jirsova

**Affiliations:** 1 Laboratory of the Biology and Pathology of the Eye, First Faculty of Medicine, Institute of Biology and Medical Genetics, Charles University and General University Hospital in Prague, Prague, Czech Republic; 2 Department of Ophthalmology, Rostock University Medical Center, Rostock, Germany; 3 Ophthalmology Department of 3^rd^ Medical Faculty and University Hospital Kralovske Vinohrady, Prague, Czech Republic; Cedars-Sinai Medical Center, UNITED STATES

## Abstract

This study aimed to determine the effect of interleukin-13 (IL13) on the stemness, differentiation, proliferation, clonogenicity, and morphology of cultured limbal epithelial cells (LECs). Human limbal explants were used to culture LECs up to the second passage (P0-P2) with or without IL13 (IL13+ and IL13-, respectively). Cells were analyzed by qPCR (for the expression of ΔNp63α, BMI-1, keratin (K) 3, K7, K12, K14, K17, mucin 4, and MKI67) and immunofluorescence staining for p63α. The clonogenic ability was determined by colony-forming assay (CFA), and their metabolic activity was measured by WST-1 assay. The results of the CFA showed a significantly increased clonogenic ability in P1 and P2 cultures when LECs were cultured with IL13. In addition, the expression of putative stem cell markers (ΔNp63α, K14, and K17) was significantly higher in all IL13+ cultures compared to IL13-. Similarly, immunofluorescence analysis showed a significantly higher percentage of p63α positive cells in P2 cultures with IL13 than without it. LECs cultures without IL13 lost their cuboidal morphology with a high nucleocytoplasmic ratio after P1. The use of IL13 also led to significantly higher proliferation in P2, which can be reflected by a higher ability to reach confluence in P2 cultures. On the other hand, IL13 had no effect on corneal epithelial cell differentiation (K3 and K12 expression), and the expression of the conjunctival marker K7 significantly increased in all IL13+ cultures compared to the respective cell culture without IL13. This study showed that IL13 enhanced the stemness of LECs by increasing the clonogenicity and the expression of putative stem cell markers of LECs while maintaining their stem cell morphology. We established IL13 as a culture supplement for LESCs, which increases their stemness potential in culture, even after the second passage, and may lead to the greater success of LESCs transplantation in patients with LSCD.

## Introduction

The corneal epithelium undergoes continuous renewal throughout life but has a limited capacity to renew itself [1]. Maintenance of the epithelium is provided by a population of limbal epithelial stem cells (LESCs), which reside in the basal layer of the limbus, the transition zone between the cornea and the conjunctiva [2]. LESCs are slow cycling and divide asymmetrically to self-renew and differentiate to maintain the corneal epithelium [3,4]. The limbus provides a unique environment—niche, which is morphologically complex with dense innervation and vascularization [5,6]. The niche of stem cells plays a crucial role in cell division, proliferation, differentiation, migration, and maintaining their stemness. It consists of cells, the extracellular matrix, cytokines, and growth factors [7]. As already demonstrated, various growth factors could induce LESCs proliferation and migration [8–11], regulation of apoptosis [12], or differentiation [13]. Because the properties of stem cells are unique, it is challenging to mimic their environment outside the niche. Therefore, the usage of LESCs for a more prolonged culture and its possible application in treatment are limited.

It is crucial for scientific and therapeutic use to search for endogenous factors that could prolong the culture of LESCs. Supplementation of culture medium with NGF extended the life span of LESCs cultures *in vitro* and increased the expression of LESCs’ putative markers *ΔNp63α* and *ABCG2* [14]. The expression of the *p63* gene is crucial for the stemness of LESCs [15]. While *TP63* transactivated isoforms (TAp63) participate in senescence and metabolism [16], the β and μ isoforms of *ΔNp63* gene play a role in epithelial differentiation during corneal regeneration, and the α variant is responsible for maintaining the stem/progenitor cells [15]. A higher percentage of the stem cell marker p63 in the sheets of transplanted limbal epithelial cells (LECs) was shown to be related to the success rate of transplantation in patients with limbal stem cell deficiency (LSCD) [17].

Our previous work found a positive effect of interleukin-13 (IL13) on proliferative activity and *ΔNp63α* gene expression in the conjunctival epithelium generated from limbal explants [18]. The effect of IL13 on increasing the expression of the *ΔNp63α* gene in conjunctival cultures leads to the idea of its role in maintaining stemness and using it as part of the medium for the culture of LESCs from limbal explants.

As a T helper 2-type cytokine, which regulates the responses of lymphocytes, myeloid cells, and nonhematopoietic cells [19], IL13 is considered one of the anti-inflammatory interleukins. The effect of IL13 on LESCs or stem cells in general is not widely studied. It is essential during early myelopoiesis when, together with the stem cell factor (SCF), it induces the proliferation of Lin-Sca-1+ progenitor cells and, together with the granulocyte-macrophage-SCF, enhances their colony formation [20]. Similarly as with IL4, IL13 can activate the STAT6 signaling pathway [19], which leads to increased clonogenic potential of prostate stem-like cells [21]. Recently, secreted IL13 was found to promote intestinal stem cell self-renewal by initiating the Foxp1 transcription factor expression, associated with the β-catenin signaling pathway responsible for maintaining their stemness [22]. Together with our observation of the role of IL13 in the stemness of conjunctival epithelial cells, it makes IL13 a promising stem cell research target. In this study, we investigated the role of IL13 in the stemness, differentiation, clonogenicity, and proliferation of LESCs.

## Materials and methods

### Donor corneal tissue

The study adhered to the tenets set out in the Declaration of Helsinki. Donor tissue procurement met all Czech legal requirements, including the absence of the donor in the national register of persons opposed to postmortem withdrawal of tissues and organs. On the use of the corneoscleral rim based on Czech legislation on specific health services (Law Act No. 372/2011 Coll.), informed consent is not required if the presented data are anonymized in the form. Forty corneoscleral rims were obtained from cadaver donor corneas within 24 h after death and stored in Eusol-C preservation medium (Alchimia, Padova, Italy) until transplantation. The mean donor age ± standard deviation (SD) was 59.3 ± 9.9 (range 41–75 years).

### Preparation of limbal explants and cell culture

The donor corneoscleral rims were washed three times in Dulbecco’s modified Eagle’s medium (DMEM)/F12 (1:1, GlutaMAX) containing 1% of 100× Antibiotic-Antimycotic (AA, Thermo Fisher Scientific, Waltham, MA, USA). Then each corneoscleral rim was cut into 12 pieces (approximately 2 × 3 mm) and washed three times in DMEM/F12 medium with 1% AA. Six explants were placed directly on the plastic bottom of the 24-well plate (one explant per well), and six pieces were placed on Thermanox plastic cell culture coverslips (Nunc, Thermo Fisher Scientific, Rochester, NY, USA) present on the bottom of the wells. Cultures grown on coverslips were used for immunostaining, and the explant grown on plastic for all other experiments. Each explant was covered with 50 μl of complete medium consisting of 1:1 DMEM/F12, 10% FBS, 1% AA, 10 ng/ml recombinant EGF, 0.5% insulin-transferrin-selenium (Thermo Fisher Scientific), 5 μg/ml hydrocortisone, 10 μg/ml adenine hydrochloride and 10 ng/ml cholera toxin (Sigma-Aldrich, Darmstadt, Germany) and maintained at 37˚C in 5% CO_2_. Complete medium was changed every day until cell outgrowth was seen. The tissue was then covered with 250 μl of complete medium, which was changed three times a week until the cells were 90–100% confluent. Half of the donor explants were cultured in a complete medium supplemented with 10 ng/ml recombinant human IL13 (BioLegend, San Diego, CA, USA). Similarly, after passage, cells were cultured in a complete medium with (IL13+) or without IL13 (IL13-). When primary cultures (P0) and cells after passage 1 (P1) were 90–100% confluent, cells were passaged using TrypLE Express (Gibco, Thermo Fisher Scientific). After detaching the culture, cells were seeded again on the plastic bottom or Thermanox coverslip in 24-well plates at concentrations of 1.5 × 10^4^ cells per well. The rest of the cells were stored in Trizol (Molecular Research Center, Cincinnati, OH, USA) for subsequent RNA isolation. All experiments were carried out on P0, P1, and P2 cells and repeated at least three times.

### Morphology, cell growth, and cell viability

Expanded LECs were monitored, and the beginning of cell outgrowth, confluence, cell morphology, viability and contamination by fibroblast-like cells were evaluated under an inverted microscope (Olympus CX41, Olympus, Tokyo, Japan). After each passage, cell viability was defined by staining with 0.4% trypan blue (Gibco, Thermo Fisher Scientific). Both unstained live cells and stained dead cells were counted with a hemocytometer and calculated as follows: viability (%) = live cells/ (live + dead cells) × 100. The percentage of successfully cultured rims was calculated as from how many corneoscleral rims the cell culture reached confluence to the total number of rims. The percentage of fibroblast-like cells contamination was calculated as how many cultures had contamination to the overall number of cultures. Culture is taken here as cell culture from one explant. From one rim 3 explants cultures were cultured with IL13 and 3 without. If one culture had fibroblast-like cell contamination, just other two were passaged to subsequent culture.

### Preparation of mouse 3T3 feeder layer

The 3T3 mouse fibroblasts were cultured in DMEM supplemented with 10% FBS and 1% AA and kept at 37˚C and 5% CO_2_. At 80–90% confluence, 3T3 cells were treated with 12 μg/ml mitomycin-C Kyowa (NORDIC Pharma, Prague, Czech Republic) for 2 h at 37 ˚C under 5% CO_2_ to arrest cell growth. After incubation, cells were washed with PBS three times, detached with TrypLE Express for 2 to 4 min, and seeded in a 6-well plate at a density of 3 x 10^5^ cells per well. Cells were used within 24 h of preparation.

### Colony-forming assay (CFA)

After reaching at least 80% confluence, the cell cultures were passaged to obtain a single-cell suspension. After P1 and P2, 1000 cultured cells were seeded in 6-well plates containing growth-arrested 3T3 mouse fibroblasts. All experiments were carried out at least in triplicate for each donor and condition (i.e., IL13- and IL13+). The LECs were kept at 37 ˚C and 5% CO_2_, and after 12 days of culture, colonies were fixed with cold methanol for 30 min at -20°C. Subsequently, the cells were rehydrated with PBS and stained for 5 min at 37˚C with 2% rhodamine B (Sigma-Aldrich). After that, rhodamine B was removed, and the colonies were washed with tap water until optimal staining intensity was achieved. The plates were photographed and manually computed by using Fiji image processing software (https://imagej.net/Fiji). Only bright-purple colonies were counted, small dots were excluded. The colony growth was monitored during the colony-formation phase under a microscope. The total colony-forming efficiency (CFE) (%) was calculated using the following equation:

$$CFE\;(\%)=\frac{number\;of\;colonies}{number\;of\;seeded\;cells}\;x\;100$$

### Immunocytochemistry

The cells for immunocytochemistry (ICC) cultured on Thermanox coverslips were at 90−100% confluency fixed in 4% paraformaldehyde for 20 min at room temperature. Immunocytochemical staining was performed for the p63α isotype encoded by the tumor protein P63 gene, *TP63*. After fixation, the cell membranes were permeabilized with 0.33% Triton X-100 (Sigma-Aldrich) diluted in PBS, followed by a 1 h incubation at room temperature with primary p63α antibody (Cell Signalling Technology, Danvers, MA, USA; cat. No: 4892) diluted in 0.1% bovine serum albumin. The cells were then rinsed three times in 0.5% Tween 20 and incubated for 1 h at room temperature with the Alexa Fluor 594 conjugated goat anti-rabbit IgG secondary antibody (Life Technologies, Eugene, OR, USA; cat. No. A11037). After rinsing three times in 0.5% Tween 20, followed by rinsing in PBS, cells were mounted with VectaShield-DAPI (4´ 6-diamidino-2-phenylindole) (Vector Laboratories, Burlingame, CA, USA) to counterstain nuclear DNA. Cell samples were examined by fluorescence microscopy (Nikon ECLIPSE Ni-U, Nikon) at ×100 and ×200 magnifications. At least 1000 cells were evaluated to calculate the percentage of positive cells.

### Quantitative real-time polymerase chain reaction (qPCR)

The expression of the *GAPDH* (glyceraldehyde-3-phosphate dehydrogenase), *ΔNp63α*, *BMI-1*, *K3*, *K7*, *K12*, *K14*, *K17*, *mucin 4* (*Muc4*), and *MKI67* genes was detected by qPCR. Cells were collected after each passage (P0-P2), transferred to Eppendorf tubes containing 500 μl of TRI Reagent (Molecular Research Center, Cincinnati, OH, USA), and total RNA was extracted according to the manufacturer’s instructions, followed by reverse transcription, which has been described previously [18]. Briefly, 1 μg of RNA was treated with deoxyribonuclease I (Promega, Madison, WI, USA) and used for reverse transcription (RT). First-strand complementary DNA (cDNA) was synthesized using M-MLV (Moloney murine leukemia virus) reverse transcriptase and random primers (Promega) in a total reaction volume of 25 μl.

The qPCR was performed in a CFX Connect Real-Time PCR Detection System (Bio-Rad, Hercules, CA, USA). The sequences of the primers used are summarized in Table 1. The sequence specificity of all primers was confirmed via BLAST (http://www.ncbi.nlm.nih.gov/blast/). Conventional reverse transcription PCR was performed to confirm that only a single band was obtained. The PCR products were electrophoresed on 1% agarose gels containing GelRed Nucleic Acid Gel Stain (Biotium, Fremont, CA, USA). The qPCR parameters included initial denaturation at 95 ˚C for 2 min, 40 cycles of denaturation at 95 ˚C for 5 s, and annealing at 60 ˚C for 30 s. Fluorescence was monitored at 55 to 95 ˚C at 0.5 ˚C intervals for 5 s. Each experiment was carried out in triplicate. A relative quantification model was used to calculate the expression of the target gene expression compared to *GAPDH*, used as the endogenous control.

**Table 1:** Primers used for quantitative real-time PCR.

Gene (human)	Sequence (5′→3′)	GenBank accession number	Product size (bp)
*GAPDH*	F: GAAGGGGTCATTGATGGCAAC R: GGGAAGGTGAAGGTCGGAGTC	NM_001289746.1	108
*ΔNp63α*	F: GAGGTTGGGCTGTTCATCAT R: GAGGAGAATTCGTGGAGCTG	NM_001114980.1	174
*BMI-1*	F: GCTCGCATTCATTTTCTGCT R: ACACACATCAGGTGGGGATT	NM_005180.8	163
*K3*	F:GGATGTGGACAGTGCCTATATG R: AGATAGCTCAGCGTCGTAGAGE	NM_057088.2	106
*K7*	F: AGGATGTGGTGGAGGACTTC R: CTTGCTCATGTAGGCAGCAT	NM_005556.3	116
*K12*	F: CCAGGTGAGGTCAGCGTAGAA R: CCTCCAGGTTGCTGATGAGCE	NM_005556.3	352
*K14*	F: TTCTGAACGAGATGCGTGAC R: GCAGCTCAATCTCCAGGTTC	NM_000526.4	189
*K17*	F: GCTGCTACAGCTTTGGCTCT R: GACGGGCATTGTCAATCTGT	NM_000422.2	315
*MUC4*	F: TCCGTGTCCTGCTGGATAACC R: GTTGCGGCTCAGGAGGACTC	NM_018406.6	104
*MKI67*	F: CTTTGGGTGCGACTTGACG R: GTCGACCCCGCTCCTTTTE	NM_002417	199

### Determination of the proliferation activity

The proliferation activity of living cells was determined by the WST-1 assay, as described before [23]. In brief, the LECs (15 x 10^3^ cells/ well) were cultured in a complete DMEM medium with or without IL13 in a 96-well tissue culture plate (VWR, Radnor, PA, USA) for 7 days at 37˚C in an atmosphere of 5% CO_2_. WST-1 reagent (Roche, Mannheim, Germany) was added to each well (10 μl/100 μl of medium), and plates were incubated for another 1 h to form formazan. A formazan-containing medium (100 μl) was transferred from each well into a new 96-well tissue culture plate. The absorbance was measured using a Tecan Infinite M200 (Tecan, Mannedorf, Switzerland) at a wavelength of 450 nm.

### Statistical analysis

Statistical analysis was performed with GraphPad Prism (GraphPad Software, La Jolla, CA, USA). Descriptive statistics are reported as N (number of values), mean ± SD, or the median with quartile range. Data sets were analyzed by Mann–Whitney U nonparametric test. P-values < 0.05 were considered statistically significant.

## Results

### Limbal epithelial cell growth and morphology

**Fig 1:**
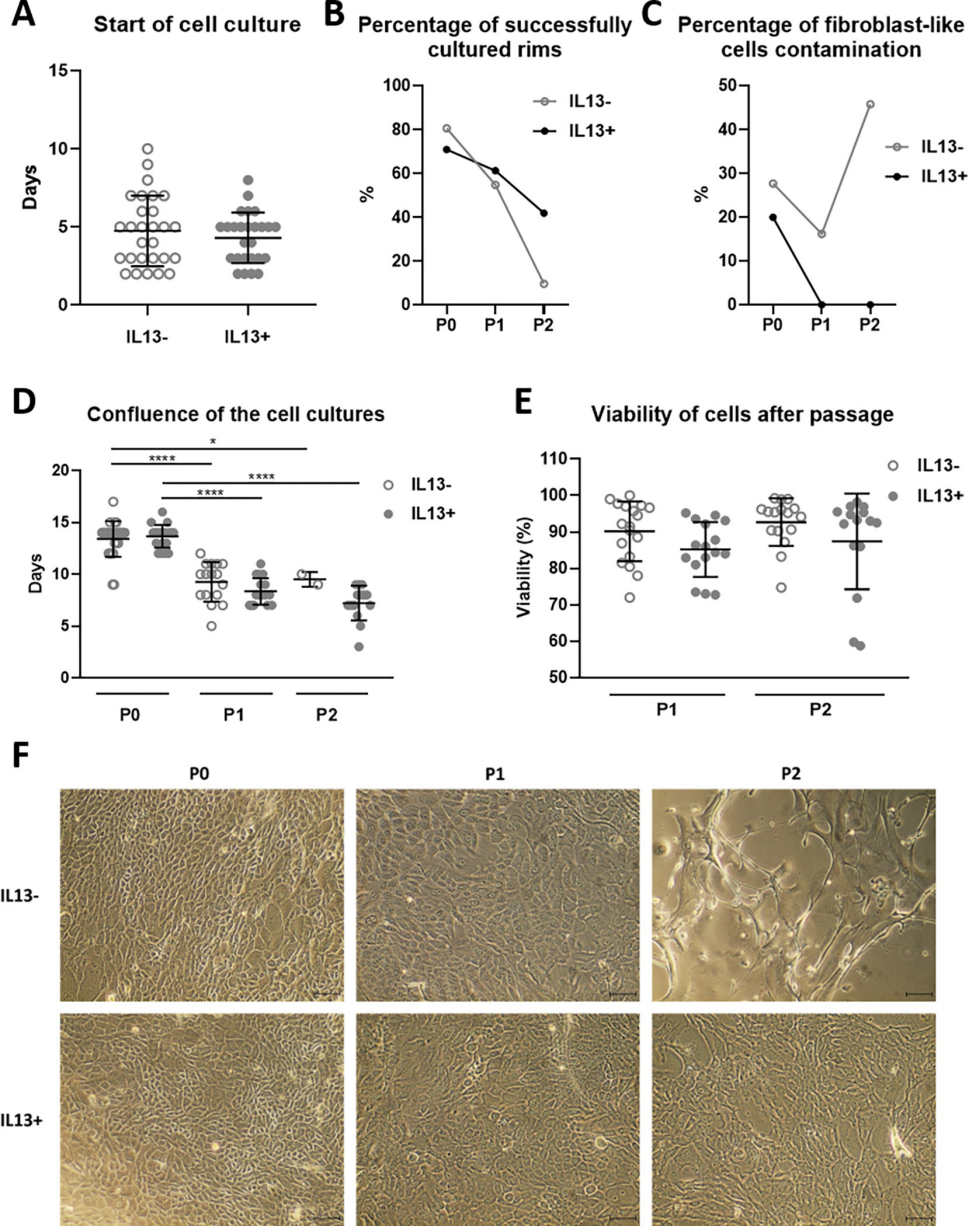
The growth and morphology of IL13- and IL13+ cell cultures. (A) The beginning of the outgrowth of LEC cultures from day 0 (D) to reaching full confluence in P0, P1, and P2 cultures. (B) The percentage of successfully cultured corneoscleral rims. (C) The percentage of fibroblast-like cells contamination in cell cultures. (E) Percentages of cell viability after the first and second passages. (F) Cell morphology was observed at the end of cultivation of P0, P1, and P2 cultures under an inverted phase-contrast microscope. Scale bars: 50 μm.

LECs P0 cultures started to outgrow from limbal explants mostly on the fifth day, and 90–100% confluence was achieved after 14 days regardless of the presence of IL13 (Fig 1A). P1 and P2 cultures reached confluence significantly earlier than P0, but there was no difference whether the cells were cultured with or without IL13 (Fig 1D). The cell viability after passaging was comparable between the cultures (Fig 1E). The percentage of successful cultures decreased throughout the cell culture from P0 to P1 and P2 (Fig 1B). The most noticeable difference was after P2, where 9.7% of IL13- cultures reached confluence, compared to 42% in the IL13+ group. This fact is also reflected in the morphology of cells. Typical cuboidal morphology of LECs with a high nucleocytoplasmic ratio was visible in all cultures, except P2 IL13- cultures, where cells were more flattened or fibroblast-like morphology with a low nucleocytoplasmic ratio (Fig 1F). The contamination by fibroblast-like cells was higher in the P0 group without than P0 with IL13 (27.7% and 20% respectively). Cell cultures without IL13 had a low percentage of contamination in P1 (16.2%) but increased in P2 (45.8%). On the contrary, the fibroblast-like cell contamination was not observed in P1 and P2 cultures with IL13 (Fig 1C).

### CFA

**Fig 2:**
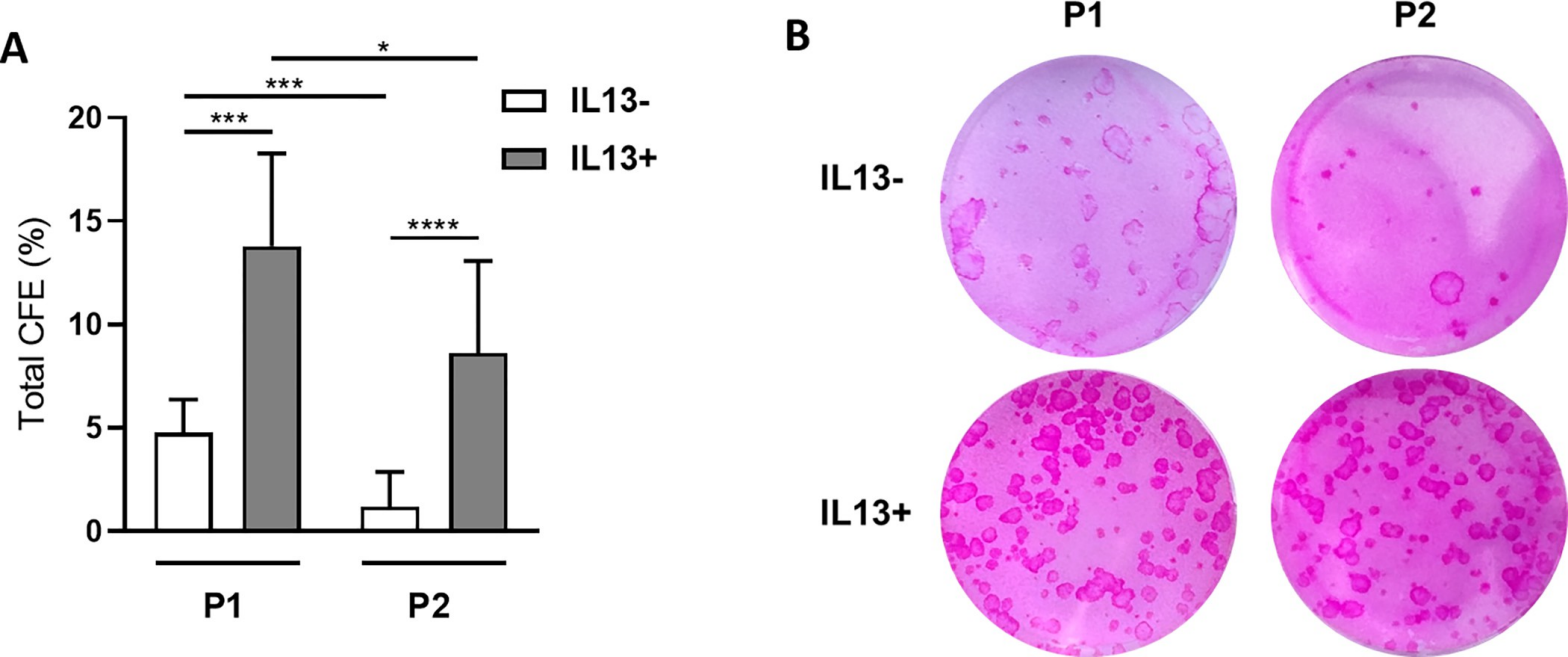
Comparison of total CFE. After the first and second passages (P1 and P2), cells were cultured with growth-arrested 3T3 mouse fibroblasts to compare their growth capacity under IL13- and IL13+ conditions. (A) Distribution of total CFE percentages of the P1 and P2 groups. Each bar represents the mean ± SD from 7 to 13 determinations. The asterisks represent a statistically significant difference between the examined groups (*P ≤ 0.05, ***P ≤ 0.001, ****P ≤ 0.0001). (B) The colonies were stained with 2% rhodamine B.

The CFA was performed for P1 and P2 cultures (Fig 2). A significantly higher growth potential was observed in the P1 and P2 IL13+ groups (mean 13.79% and 8.63% respectively) than the P1 and P2 IL13- cultures (mean 4.78% and 1.19% respectively), P<0.001 and P<0.0001 respectively. The decrease of the number of colonies was between both passages and IL13 groups, more significant in IL13- than in IL13+ cultures (P< 0.001 vs. P<0.05).

### Expression of limbal stem cell markers

**Fig 3:**
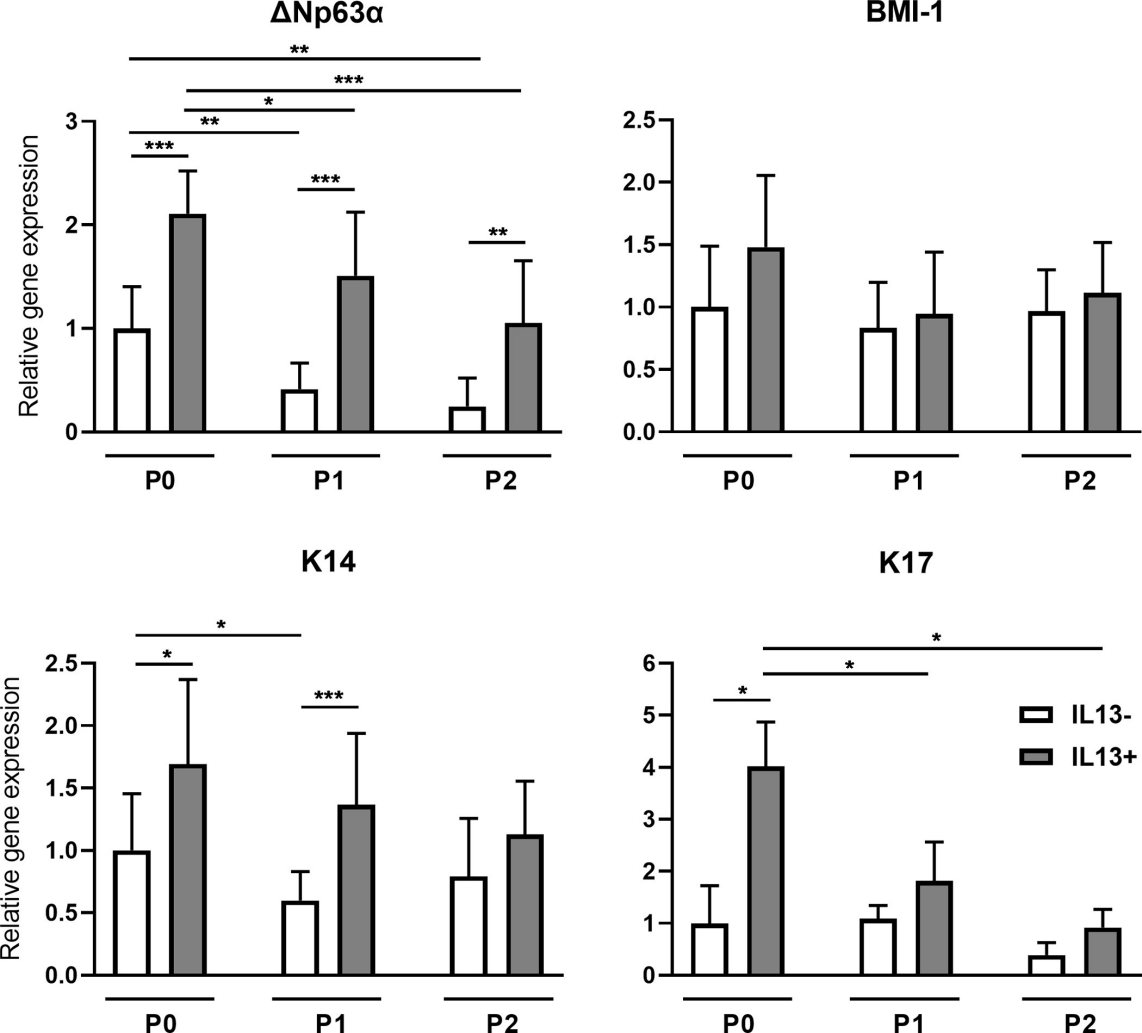
Relative gene expression of *ΔNp63α*, *BMI-1*, *K14*, and *K17* in IL13- and IL13+ cell cultures. Cells originating from limbal explants (P0) and passaged cells (P1 and P2) were analyzed at the end of the culture for *p63α*, *BMI-1*, *K14*, and *K17* gene expression by qPCR. Each bar represents the mean ± SD of three to ten determinations. The asterisks represent statistically significant difference between the examined groups (*P < 0.05, **P ≤ 0.01, ***P ≤ 0.001).

The *ΔNp63α* gene expression was significantly higher in all cultures (P0, P1, and P2) with IL13 compared to the cells cultured without it (P<0.001, P<0.001, P<0.01, respectively). A consistent decrease of *ΔNp63α* gene expression was observed during the LECs culture with significance between P0-P1 and P1-P2 cultures in the IL13- (both P<0.01) and IL13+ (P< 0.05, P<0.001 respectively) groups. A slightly higher expression of the *BMI-1* gene was observed in P0 IL13+ cultures compared to IL13- cells with no statistical significance. The *BMI-1* expression values in P1 and P2 cultures were comparable. The gene expressions of *K14* were significantly higher in the IL13+ groups P0 (P<0.05) and P1 (P<0.001) than controls without IL13. The expression decreased significantly between P0 and P1 cultures without IL13 (P<0,05). A decreased *K17* gene expression tendency was found throughout the cell culture with statistical significance between the P0 IL13+ culture and the P1 and P2 IL13+ groups, respectively (both P<0.05). The expression of the *K17* gene was significantly higher in samples with IL13 in P0 (P<0.05) compared to samples without IL13 (Fig 3).

### Immunocytochemical staining for p63

**Fig 4:**
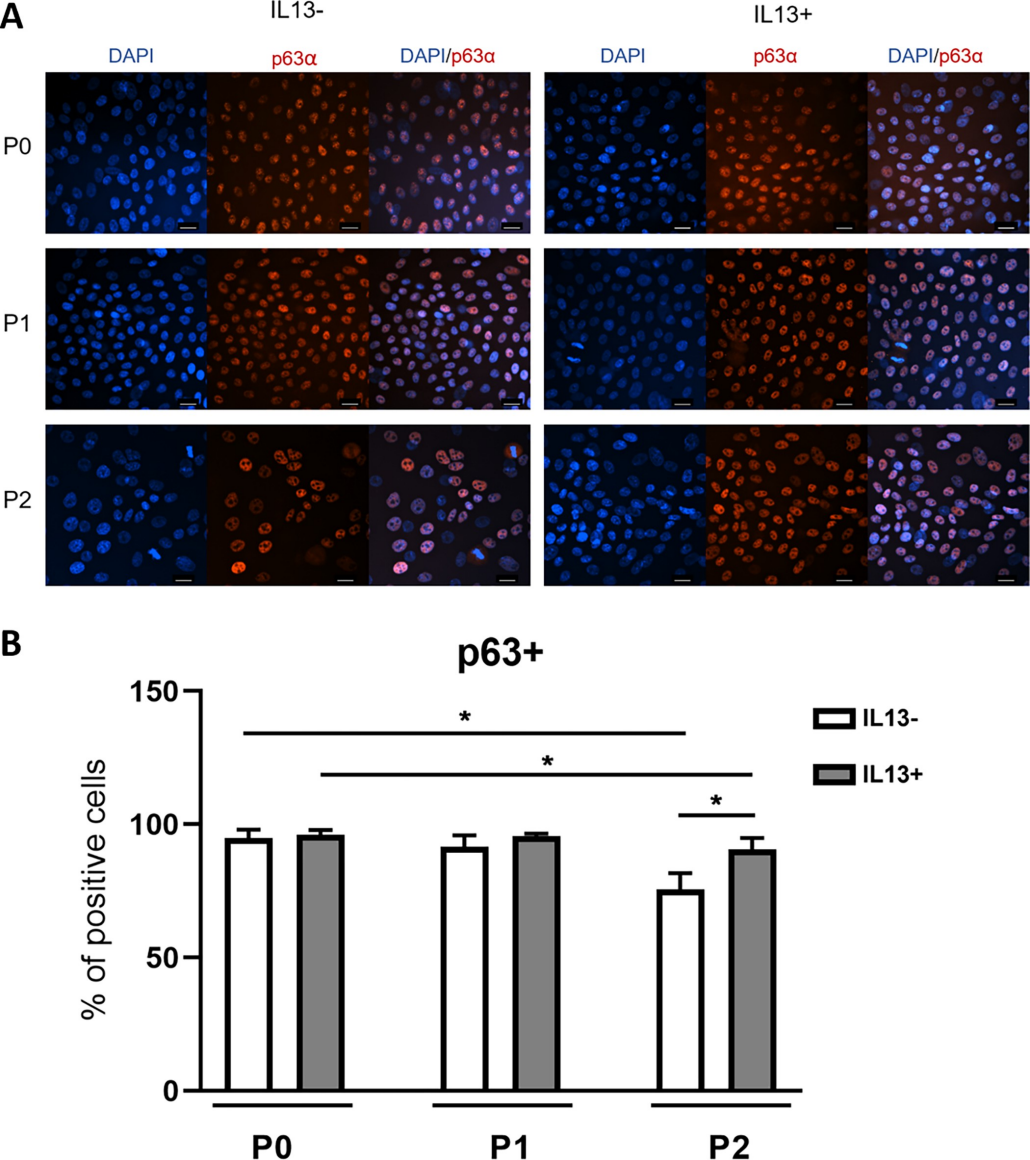
Immunostaining for the putative limbal stem cell marker p63α in IL13- and IL13+ cell cultures. (A) Cells were analyzed by immunofluorescent staining for p63α (red) at the end of P0-P2 cultures. Nuclei were counterstained with DAPI (blue). Scale bars: 20 μm. (B) Distribution of percentages in the P0, P1, and P2 groups for p63α staining. Each bar represents the mean ± SD of three to eight determinations. The asterisks represent a statistically significant difference between the examined groups (*P ≤ 0.05).

Cells positive for the p63 protein were detected in all cultures and conditions (Fig 4A). The percentage of p63 positive cells significantly decreased during cell culture without IL13 (mean values 94.86%, 91.65%, and 75.55% for P0, P1, and P2 cultures, respectively; P<0.05). Cell cultures with IL13 had a similar expression of p63 in all passages (mean values 96.23%, 95.54%, and 90.69% for P0, P1, and P2 cultures, respectively). A significantly higher percentage of p63+ cells were measured in P2 IL13+ compared to P2 IL13- culture (P<0.05) (Fig 4B).

### LECs proliferation and metabolic activity

**Fig 5:**
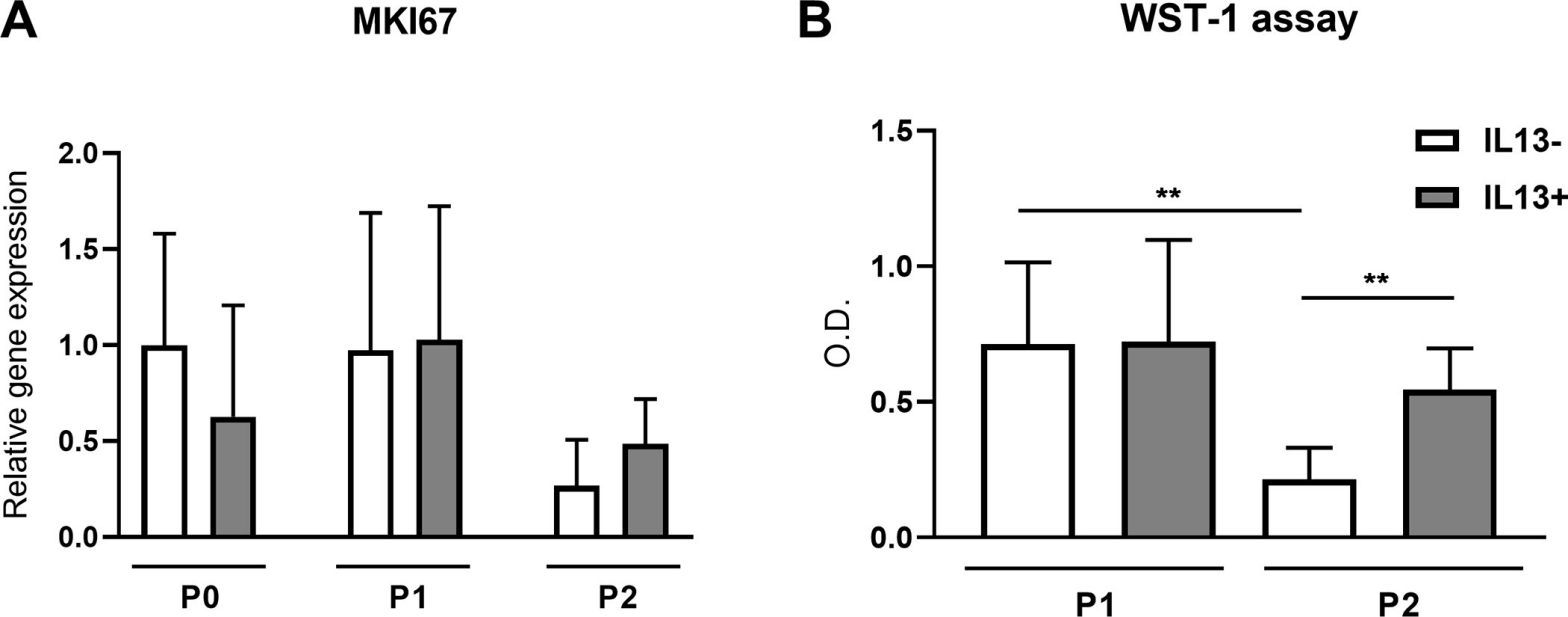
Relative gene expression of *MKI67* and WST-1 assay in IL13- and IL13+ cell cultures. (A) Cells originating from limbal explants (P0) and passaged cells (P1 and P2) at the end of culture were analyzed for *MKI67* gene expression by qPCR. Each bar represents the mean ± SD of five to ten determinations. (B) Measurement of cell proliferation after the first and second passages (P1 and P2). The WST-1 reagent was added to the cell cultures for 1 h to form formazan. The absorbance was measured at a wavelength of 450 nm via optical density. Each bar represents the mean ± SD of eight to ten determinations. The asterisks represent a statistically significant difference between the examined groups (**P ≤ 0.01).

The proliferation of cultured LECs was determined by gene expression analysis of *MKI67* and WST-1 assay (Fig 5). No significant difference was observed between the evaluated groups for *MKI67* gene expression. No difference was found between IL13+ and IL13- groups in P1, but significantly higher proliferation was determined in P2 IL13+ compared to P2 IL13- (P<0.01) according to WST-1 assay. The significant decrease of the proliferation activity was measured between P1 and P2 cultures without IL13 (P<0.01).

### Presence of differentiation markers

**Fig 6:**
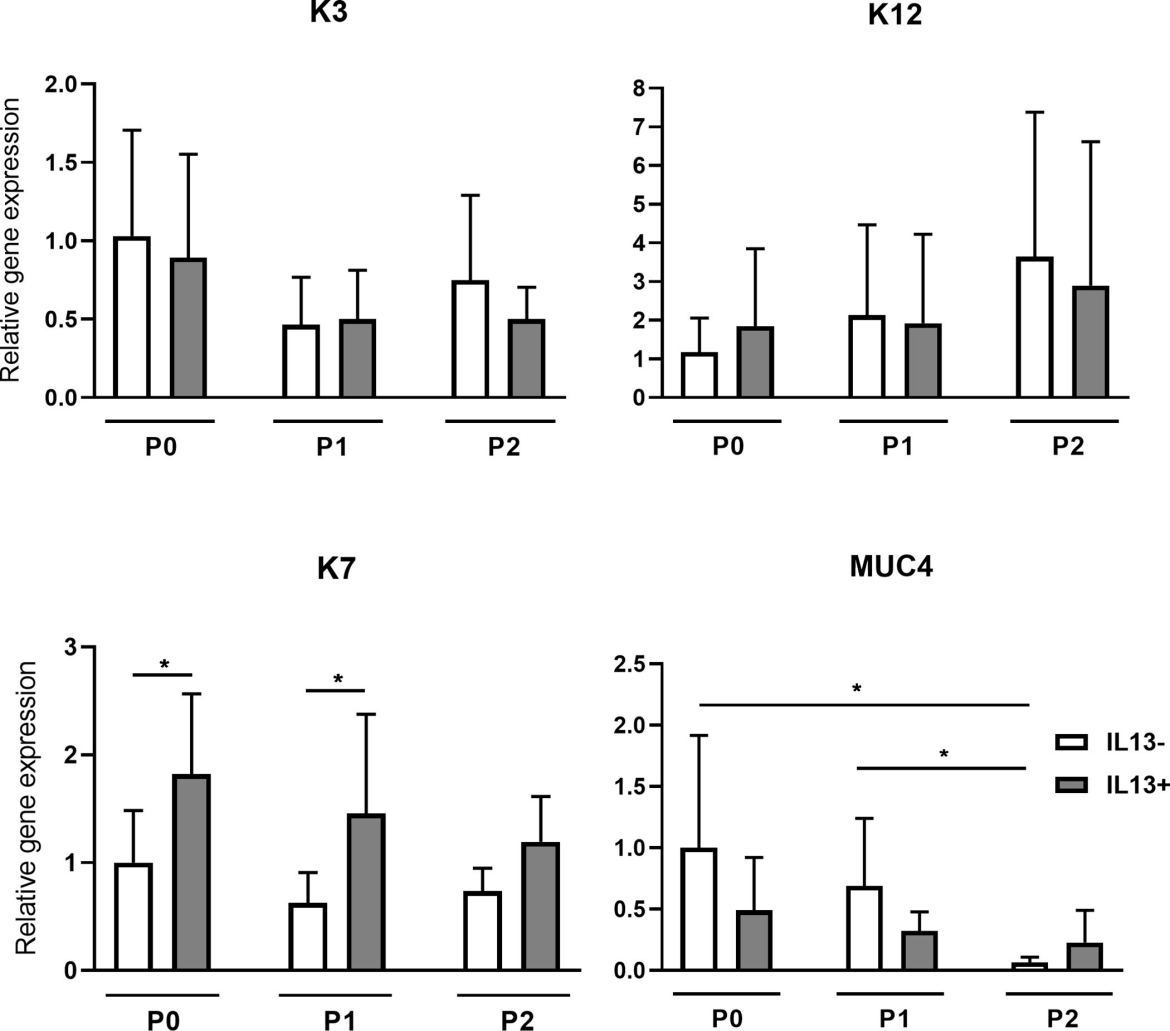
Relative gene expression of *K3*, *K12*, *K7*, and *MUC4* in IL13- and IL13+ cell cultures. Cells originating from limbal explants (P0) and passaged cells (P1 and P2) at the end of culture were analyzed for the expression of *K3*, *K12*, *K7*, and *MUC4* genes by qPCR. Each bar represents the mean ± SD of four to ten determinations. The asterisks represent a statistically significant difference between the examined groups (*P < 0.05).

*K3* and *K12* genes specific for the corneal epithelium were expressed in all groups at very low levels with no difference and significance. Conjunctival *K7* gene expression was higher in all IL13+ cell passages compared to IL13- cultures, significantly in P0 and P1 (both P<0.05). The *MUC4* gene was expressed in all groups with a significant decrease between passages P0 and P2 and between P1 and P2 in IL13- conditions (P<0.05) (Fig 6).

## Discussion

In this study, we explore the potential of IL13 to improve the properties of cultured LECs, particularly in terms of their stemness and use for the treatment of LSCD. Our results showed that IL13 significantly increases stemness of LECs after P1 and P2, as shown by the CFA, by the expression of putative stem cell markers (*ΔNp63α*, *K14*, and *K17*), and by immunocytochemistry (p63α). Besides the increase in stemness, there was no change in the morphology, but cell proliferation was significantly higher after P2 cultures with IL13 compared to without IL13.

The clonogenic activity of our LESCs cultures without IL13 was about 5% after the first passage, decreasing to 1% after the second passage. This percentage was higher than the clonogenic activity in conjunctival cultures or limbal explants in previous studies [18,24]. Cultures with IL13 had significantly higher clonogenic capacity after both passages than the related passage of cultures without IL13. The higher clonogenic activity of IL13+ cultures corresponds to increased gene expression of the putative stem cell marker p63α. Previously, it was reported that coexpression of C/EBPΔ, BMI-1, and ΔNp63α identified mitotically quiescent limbal stem cells, which generate holoclones in culture [25].

Furthermore, human holoclone-forming cells have been shown to be only located in the limbus [26,27], and that more than 3% of p63 positive cells from total clonogenic cells in the culture led to successful transplantation in patients with LSCD. Therefore, clonogenic activity is an important indicator of stem cells, as the competence for the continued proliferation of stem cells in tissues is required for normal tissues’ continued integrity and function [28]. The time-dependent decrease in clonogenicity is consistent with the observation of other studies; a 4-day culture of LECs led to differentiation and loss of almost all the capacity for colony formation [14]. The loss of clonogenic properties in transient amplifying cells during culture was also found by Pellegrini et al. [26]. Moreover, a decreased number of cells with p63 stemness factor was observed during a more extended culture of LECs from limbal explants [29]. It looks like a typical stem cell process. The clonogenic activity decreased rapidly in our LECs cultures from 5% after P1 to 1% after P2. The preservation of clonogenicity and stemness due to IL13 also corresponds to the cuboidal morphology of cultured cells that had a high nucleocytoplasmic ratio during all cultures. On the contrary, LESCs cultures without IL13 lost their morphology after the second passage, consistent with other works [14,18]. Fibroblast-like cells contamination was lower or not present in cultures with IL13, and the epithelial phenotype was predominant compared to cultures without IL13. Still, more studies are necessary to analyze whether IL13 inhibits fibroblasts’ outgrowth directly or the lower contamination is because of higher stemness in the IL13 cultures.

Our gene expression analysis showed that LECs culture with IL13 increased the expression of K14, K17, and most importantly, ΔNp63α stem cell markers, which are generally used to indicate stemness in LESCs cultures [30–33]. However, according to immunohistochemistry, the percentage of p63 positive cells showed a significant difference between cultures with or without IL13 only in P2, in which a slight increase in p63 positive cells was observed in IL13+ cells compared to IL13- culture. The discrepancy between qPCR and immunostaining findings has been previously found [34,35]. It can be explained by immunostaining analysis showing only the number of positive cells and not the amount of protein expressed. In addition, the antibody is less specific than the qPCR primers due to the six isoforms of the *p63* gene and the minor differences between them.

Generally, LESCs are slow-cycling during homeostasis and highly proliferative in case of injury [36]. Our gene expression analysis of the *MKI67* gene, which is involved in cell proliferation, showed weak expression and no difference between cultures with or without IL13 in all passages. Joyce et al. showed in the study of the expression of MKI67 in the human cornea by indirect immunofluorescence localization that most LECs did not present this marker of cell proliferation [37]. A similar observation was found in the study of limbal explant cultures, in which epithelial cells were positive for MKI67 in the first week of culture, but most expression disappeared by 3 weeks when cells migrating from explants reached confluence [29]. They hypothesized that MKI67 expression indicates the proliferative status of cells in a certain time but not the proliferation capacity of cells, as they showed a decrease in MKI67 staining in cells from confluent cultures [29]. This is in accordance with the weak gene expression of MKI67 in our cultures, as we measured the expression after the passage when cells were about to reach the confluence. Therefore, we also used another measurement of cell proliferation, the WST-1 assay, which showed no difference in cell proliferation between P1 and P2 IL13+ cultures. However, a substantially higher proliferation of IL13+ than IL13- cultures after the second passage indicates their potential to be used as a graft even after passage. This observation follows the weak potential of LECs after P2 without IL13 to reach confluence.

The gene expression of corneal epithelial markers K3 and K12 did not differ between the IL13- and IL13+ cultures. The expressions of these genes were weak, and we did not observe any changes leading to differentiation into the corneal epithelial phenotype. In contrast to our findings, some works showed 60–80% positivity of K3 and K12 in LESCs cultures [24,38]. The low expression of corneal epithelial factors can be explained as a positive effect of IL13, as the culture cells did not differentiate into the corneal epithelial phenotype but retained their stemness. Nevertheless, it is unclear whether cells with a differentiated corneal epithelial phenotype in the graft are more important for transplant success than the presence of a niche with cells with varying degrees of differentiation [39].

Here we show that culture influenced by IL13 led to a higher differentiation into conjunctival phenotype, as we measured a higher gene expression of the *K7* gene in all cultures (P0-P2). K7 is considered a marker of the conjunctival epithelium [40]. The same differentiation potential of IL13 was observed in conjunctival cultures from limbal explants [18]. Interestingly, the expression of another conjunctival marker, MUC4, was not affected by the addition of IL13 and decreased significantly during passages. Because conjunctival cells have also been used for the successful treatment of LSCD, the presence of K7 is unlikely to adversely affect the quality of the graft. Our previous results with conjunctival epithelial cells showed that partial differentiation to the conjunctival phenotype was expected [18]. However, unlike corneal conjunctivalization, which includes a vascularized pannus that overgrows on the cornea in LSCD-affected eyes, these transplanted conjunctival epithelial cells do not have any associated fibrous or vascular tissue [41].

IL13 is essential during early myelopoiesis. However, its role in the stemness of LESCs or other cell types was unknown. The first findings were made in conjunctival cell cultures from limbal explants, where IL13 maintained the stemness of the cultures by increasing their clonal capacity and p63α expression [18]. The detailed signaling pathway for this mechanism is unclear. A similar observation of maintaining stemness via IL13 –IL13R (IL13 receptor) was recently described in intestinal stem cells (ISCs) [22]. They found that the circular RNA molecule circPan3 bounds the mRNA encoding the cytokine IL13R subunit IL13Rα1 in ISCs to increase the stability of the receptor. IL13 then binds to IL13R, which initiates the expression of the transcription factor Foxp1. Foxp1 is associated with the β-catenin signaling pathway, causing activation of this pathway and maintenance of ISCs. The deletion of circPan3 in ISCs led to impaired stem cell self-renewal capacity and regeneration of the intestinal epithelium [22]. IL13 can induce through IL4Rα present in the human corneal epithelial cells the expression of various genes, such as *HAS3*, encoding hyaluronan synthases; hyaluronan in the extracellular matrix has been shown to control epithelial proliferation and regeneration [42,43]. The Wnt/β-catenin signaling is also present in the ocular surface epithelium and plays an important role in regulating LESCs proliferation [41]; further investigation of this signaling pathway would better explain the whole mechanism of IL13 in LESCs.

This study showed that IL13 enhanced the stemness of LESCs by increasing the clonogenicity and the expression of putative stem cell markers of LECs while maintaining their stem cell morphology. We established IL13 as a culture supplement for LESCs, which increases their stemness potential in culture, even after the second passage, and may lead to greater success of LECs transplantations in patients with LSCD.
